# The relationship between dlPFC activity during unpredictable threat and CO_2_-induced panic symptoms

**DOI:** 10.1038/s41398-017-0006-5

**Published:** 2017-11-30

**Authors:** Nicholas L. Balderston, Jeffrey Liu, Roxann Roberson-Nay, Monique Ernst, Christian Grillon

**Affiliations:** 1 Section on Neurobiology of Fear and Anxiety, National Institute of Mental Health, National Institutes of Health, Bethesda, MD USA; 2 Virginia Institute for Psychiatric and Behavioral Genetics, Department of Psychiatry, Virginia Commonwealth University, Richmond, VA USA

## Abstract

Panic disorder is characterized by sudden, repeated, and unexpected attacks of intense fear and overwhelming anxiety about when another attack may strike. Patients with panic disorder and healthy individuals with a history of panic attacks show a hypersensitivity to unpredictable threats, suggesting a possible link between panic and sustained anxiety. The purpose of this study was to determine the degree to which induced symptoms of panic relate to fear and anxiety, as well as activity in the neural systems that mediate and regulate these affective states. Psychological and physiological symptoms of panic were assessed during an 8-min 7.5% CO_2_ challenge task. Psychological, physiological, and neural symptoms of fear and anxiety were measured during two sessions (one psychophysiology and one functional magnetic resonance imaging where subjects experienced several blocks of no threat (N), predictable shock (P), and unpredictable shock (U; NPU threat task). We used a principle component analysis to characterize panic susceptibility (PS), and found that PS significantly predicted dorsolateral prefrontal cortex (dlPFC) activity to the unpredictable cue during the NPU threat task. When examining the weighted beta coefficients from this analysis, we observed that self-reported fear/anxiety during the CO_2_ challenge negatively loaded onto dlPFC activity during the NPU task. Consistent with this observation, dlPFC activity during the unpredictable cue was also negatively correlated with anxiety during the NPU sessions. Together, these results suggest that panic symptoms and anxiety are regulated by the same prefrontal cognitive control system.

## Introduction

Panic disorder is characterized by sudden, repeated, and unexpected attacks of intense fear and anxiety^1^. Not only do individuals with panic disorder suffer from these paralyzing attacks, but they also express intense worry and overwhelming anxiety about when another attack may strike^1^. Although this disorder may profoundly impact the quality of life of the affected individuals, we know little about the etiology of this disorder, or the neural and cognitive systems that maintain and regulate panic symptoms^2^.

Many symptoms of panic attacks, such as breathing problems, dizziness, and numbness, can be traced to the respiratory system^2^. Accordingly, elevated CO_2_ blood levels are thought to contribute to panic attacks^2^. To study panic attacks, researchers have developed a CO_2_ challenge to experimentally induce panic symptoms. During this challenge, enriched air (5–7.5% CO_2_) is inhaled for up to 20 min, resulting in elevated symptoms of anxiety and panic, especially in those susceptible to panic disorder^3–7^. Although effective for identifying panic susceptibility (i.e., elevated psychophysiological arousal and intense feelings of fear/anxiety in response to the CO_2_ administration), little is known about how CO_2_-induced panic symptoms relate to fear and anxiety.

Responses to threats are heterogeneous. One recognized distinction is that between fear, an emergency reaction to a proximal and/or predictable threat, and anxiety, a more sustained state of apprehension in response to a distal and/or unpredictable threat^8,9^, which are mediated by distinct core neural systems^9^. Acute fear is supported by the amygdala, while sustained anxiety is supported by the bed nucleus of the stria terminalis (BNST)^9^. There is evidence to suggest that panic symptoms may be more related to sustained anxiety than to acute fear. For instance, panic disorder patients^10,11^ and individuals with a history of panic attack^12^ show hypersensitivity to unpredictable but not predictable threats, and patients with Urbach–Wiethe disease can experience panic attacks, even without a healthy amygdala^13,14^. These results suggest that CO_2_-induced panic symptoms may not be mediated by the canonical acute fear circuit; however, less is known about CO_2_-induced panic and sustained anxiety. The so-called “NPU threat task” is the gold standard for experimentally studying fear and anxiety in humans^15^, and is part of the Research Domain Criteria matrix put forth by the National Institute of Mental Health (NIMH)^16^. The NPU threat task consists of periods of No shock, Predictable shocks, and Unpredictable shocks. The predictable condition can evoke acute fear, while the unpredictable condition can evoke anxiety.

In spite of these core neural differences, fear and anxiety also engage common neural systems. For instance, both acute fear and sustained anxiety recruit a large network of structures important for emotional expression (fear network; FN), including the dorsal anterior cingulate cortex/dorsomedial prefrontal cortex (dmPFC), the anterior insula, and the thalamus^17–24^. Similarly, both fear and anxiety disengage regions of the default mode network (DMN), which may play a key role in planning and self-referential processing^25–28^. Finally, fear and anxiety are both regulated by similar cognitive systems^29–42^. For instance, tasks that engage regions of the cognitive control network, such as the dorsolateral prefrontal cortex (dlPFC), reduce fear and anxiety possibly through implicit emotion regulation^43–47^. Therefore, although the core systems mediating fear and anxiety are different, these emotional states rely on similar auxiliary systems.

Because the amygdala may not be necessary for panic, it is clear that panic, like anxiety, is mediated by a core system separate from the fear system^13,14^. Given that individuals with panic disorder or a history of panic attack show heightened anxiety to unpredictable threat^10,11^, one might hypothesize that anxiety and panic share common core systems. In addition, the contributions of these other, auxiliary, systems to the expression of panic symptoms are less clear. Therefore, one might hypothesize that the ability to engage, disengage, and regulate activity in these canonical networks (e.g., FN, DMN, cognitive control, etc.) may represent a core component of these similar emotional states, and thus the disorders are characterized by overexpression of these states^48,49^. Therefore, the purposes of this study are to (1) determine the degree to which panic-susceptible individuals show elevated measures of fear and anxiety during the NPU threat task, and (2) determine the degree to which panic-susceptible individuals show abnormal reactivity in the neural systems that mediate or regulate fear and anxiety. Accordingly, we recruited healthy subjects, and screened them for panic susceptibility using a maintained 7.5% CO_2_ challenge, and then used their self-report and physiological responses to predict behavioral and neural responses in the NPU threat task.

## Materials and methods

### Participants

Eighty-four healthy, right-handed volunteers from the Washington DC area were recruited by advertisements, word of mouth, and medical referrals into the present study. Sample size was maximized based on available scanning resources, and it surpassed the minimum sample size needed to obtain the effects described in Schmitz and Grillon^15^. Potential participants were given a comprehensive evaluation by the clinical staff at the National Institute of Health Clinical Center in Bethesda, MD. Participants were excluded if they had (1) current or past history of any axis I psychiatric disorder as assessed by SCID-I/NP (2) first-degree family history of mania, schizophrenia, or other psychoses, (3) current or past history of any psychotropic or illicit drug use confirmed by a negative urine screen, (4) brain abnormalities on MRI as assessed by a licensed radiologist, or (5) medical conditions or that interfered with the objectives of the study.

Nine participants withdrew or could not be scheduled for all three experimental sessions. Two participants were excluded on the basis of performance (e.g., falling asleep or not paying attention), six participants were excluded on the basis of contaminated data sets (e.g., movement, excessive noise, etc.), and 4 participants were excluded on the basis of missing self-reports, leaving 63 completers (28 female; *M* (SD): 27 (5.7) yo). All participants gave written informed consent approved by the NIMH Combined Neuroscience Institutional Review Board and received financial compensation.

### Procedure

#### Overview

The purpose of this study was to identify relationships between panic susceptibility and psychological, psychophysiological, and neural measures of fear and anxiety. To characterize panic susceptibility, we administered a 7.5% CO_2_ challenge and collected several psychological and psychophysiological measures of panic symptomology. To characterize the psychological and psychophysiological aspects of fear and anxiety, we administered a laboratory version of the NPU threat task, during which shocks are delivered predictably or unpredictably. Fear was defined as the response to the cue during the predictable blocks, and anxiety was defined as the response during the unpredictable blocks compared to the neutral blocks. Finally, to characterize the neural aspects of fear and anxiety, we administered a functional magnetic resonance imaging (fMRI) version of the NPU threat task. The study consisted of two visits on separate days. During the laboratory visit, subjects completed the CO_2_ challenge and NPU threat task. During the MRI visit, subjects completed the NPU threat task without startle probes. Separate NPU visits for psychophysiology and fMRI recordings were conducted because the hardware to administer the white-noise probes or collect the appropriate psychophysiological measures (i.e., the acoustic startle response) in the MRI scanner were not available. Visit and NPU block orders were counterbalanced across participants. Additional methodological details can be found in the Supplementary Methods.

#### CO_2_ challenge procedure

Subjects were seated and affixed with a silicone facemask (Hans Rudolph Inc.) that covered their mouth and nose. The facemask was connected through gas-impermeable tubing to a non-diffusing gas bag (Hans Rudolph Inc.), via a three-way stop cock, which allowed the researcher to manually switch from room air to the 7.5% CO_2_ mixture. Once fitted with mask, participants breathed 5 min of room air (Pre-CO_2_), followed by 8 min of 7.5% CO_2_ (CO_2_-inhalation), followed by 5 min of room air (recovery). The mask was removed after the recovery period. Subjects were blind to CO_2_ onset, but informed that they could withdraw at any point.

#### NPU laboratory session

Electrodes to measure the startle response and deliver the shocks were attached to the subject, and the subject was given headphones for the startle response. Next, the subjects underwent a standard startle habituation block where they received nine unsignaled white-noise presentations (used to probe the acoustic startle reflex). Afterward, a shock workup procedure was done to set the level of shock (used to induce anxiety).

The NPU task consisted of three types of blocks: Neutral (N), Predictable (P), and Unpredictable (U). During each block, an 8-s cue was presented three times. The cues were simple geometric shapes with three, four, or five sides that were colored orange (RGB color: 255, 128, 0), teal (RGB color: 0, 128, 255), or purple (RGB color: 128, 0, 255). Different cues were used for the N, P, and U blocks, and both the color and the shape of the cues were determined randomly for each subject at the start of the experiment. During the N blocks, subjects were informed that they would not receive a shock, regardless of the presence or absence of the cue. During the P blocks, subjects were informed that they could receive a shock, but only during the cue. During the U blocks, subjects were informed that they could receive a shock anytime. This information was provided both before the experiment and throughout each block via text prompts. Throughout, subjects were informed that they would receive periodic white-noise presentations (for startle measurements). Subjects were instructed to continuously rate their anxiety using an online likert-type scale.

There were two runs consisting of alternating N, P, and U blocks with the following sequences: PNUNUNP or UNPNPNU. Six white-noise probes were administered (three during the cue and three during the Intertrial Interval (ITI)). Ten shocks were randomly distributed across the predictable (during the cue) and unpredictable blocks, with five shocks occurring in each block type. The run order was counterbalanced across subjects.

#### NPU fMRI session

Subjects were prepped to go into the MRI scanner (given earplugs, situated on the scanner table, etc). Afterward, a shock workup procedure was done to set the level of shock. Once situated, subjects received a structural scan (T1), an 8-min pre-NPU resting EPI scan, 2 EPI scans during the NPU threat task, and an 8-min post-NPU resting EPI scan. The procedure for the NPU threat task was identical to that of the laboratory session, with the exception that no startle probes were presented. The resting scans were not analyzed for this study.

### Materials

For the CO_2_ challenge, we collected several psychological (Diagnostic Symptom Questionnaire (DSQ)^1^, Subjective Units of Distress Scale (SUDS)^50^) and psychophysiological (tidal lung volume (LV), capnography (CO_2_%), heart rate (HR), heart rate variability (HRV), skin conductance, and respiratory rate (RR)) measures of panic symptomology. During the NPU laboratory session we collected startle and anxiety ratings. During the NPU fMRI session we collected BOLD and analyzed the cue-evoked activity for the N, P, and U conditions. For a full discussion of the methods see the Supplementary Methods.

### Analysis

The analysis strategy was 2-fold. First, we considered the effects of each manipulation on the corresponding dependent measures. For the CO_2_ challenge we examined the change in each measure from Pre-CO_2_ period to CO_2_ period. For the NPU laboratory session, we identified behavioral measures that reflected sustained anxiety during the unpredictable blocks by calculating anxiety-potentiated startle (APS) and anxiety-potentiated ratings (APR). We also calculated behavioral measures that reflected acute fear by calculating fear-potentiated startle (FPS) and fear-potentiated ratings (FPRs). For the NPU fMRI session, we conducted a one-way ANOVA on the cue-evoked betas, corrected for multiple comparisons using cluster thresholding, and examined the post hoc pairwise comparisons at the cluster level.

Next, we examined the relationship between the measures of each experiment. The goal was to explain as much variability in the data using the fewest possible comparisons. Accordingly, we first reduced the data from each experiment using a principal component analysis (PCA), and used the component scores (regressors of interest) in a general linear model (GLM) to predict anxiety and/or panic symptoms for the two remaining experiments. Finally, to characterize each GLM we combined the PCA item loadings for each component with that component’s coefficient, yielding weighted coefficients for each item entered into the GLM. For a complete discussion of data processing and analysis, see the Supplementary Materials.

## Results

### CO_2_

**Fig. 1:**
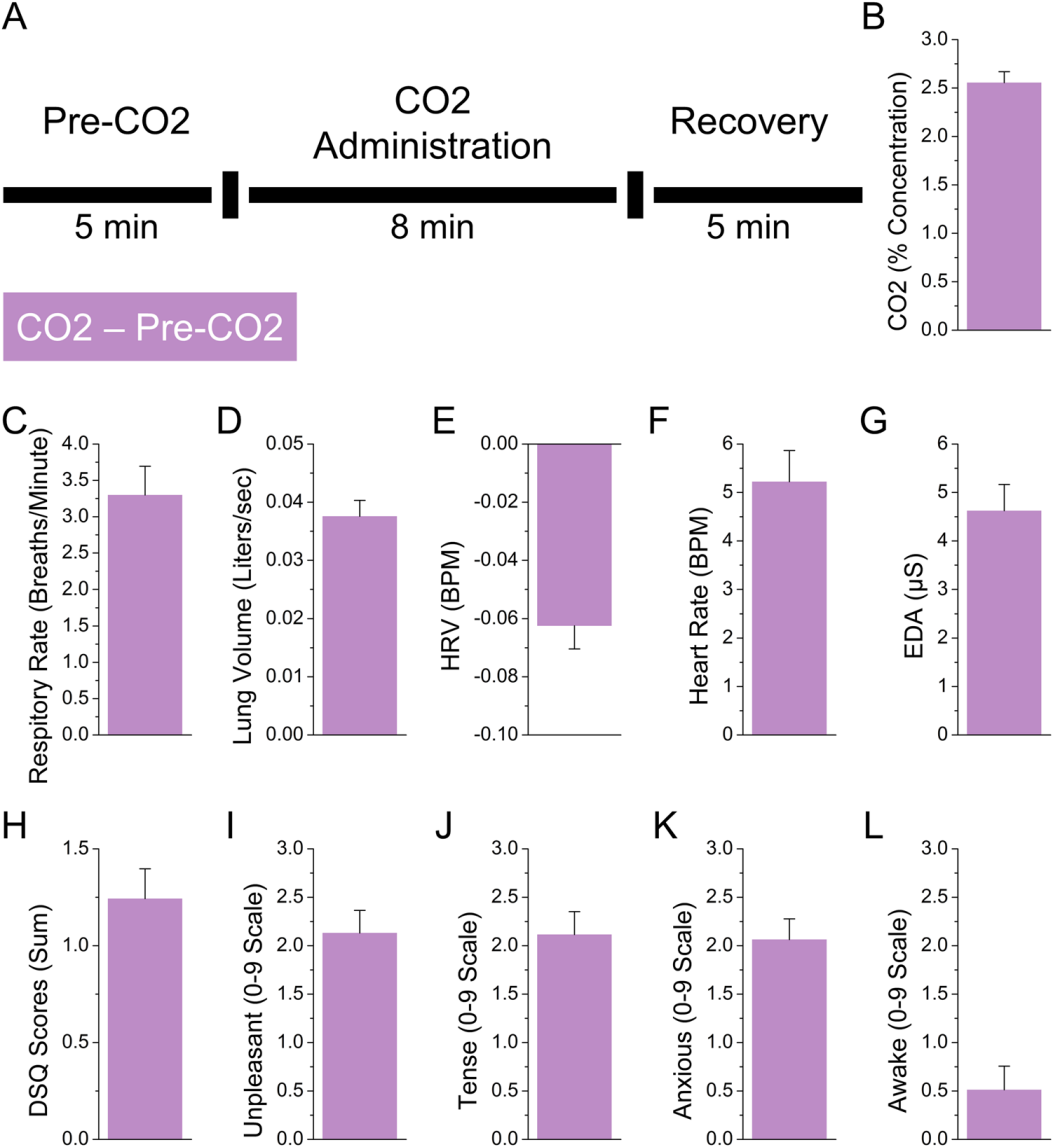
Design and primary outcome measures from the 7.5% CO_2_ challenge **a** Schematic demonstrating timeline of the CO_2_ challenge. During the pre-CO_2_ and recovery periods, subjects breathe room air. During the CO_2_ administration period, subjects breathe room air enriched with 7.5% CO_2_. **b**–**k** Physical **b**–**g** and psychological **h**–**l** symptoms of CO_2_ administration. Bars represent the mean CO_2_ − Pre-CO_2_ values ± SEM

To determine the effectiveness of the CO_2_ challenge, we created CO_2_−Pre-CO_2_ difference scores for variables reflecting: breathing (RR, LV, end-tidal CO_2_), physiological arousal (HR, HRV, and EDA), subjective panic symptoms (DSQ), and subjective emotional state (SUDS: Unpleasant, Anxious, Awake, Tense; see Fig. 1). As expected, subjects showed increased breathing (RR: t(62) = 8.24; *p* < 0.001; *d* = 1.05, LV: t(62) = 13.57; *p* < 0.001; *d* = 1.72, CO_2_: t(62) = 22.41; *p* < 0.001; *d* = 2.85), increased arousal (HR: t(62) = 8.04; *p* < 0.001; *d* = 1.02, HRV: t(62) = −7.75; *p* < 0.001; *d* = −0.98, EDA: t(62) = 8.46; *p* < 0.001; *d *= 1.07), elevated symptoms of panic (DSQ: t(62) = 8.01; *p* < 0.001; *d* = 1.02), and an increase in negative emotional state (Unpleasant: t(62) = 9.04; *p* < 0.001; d = 1.15, Anxious: t(62) = 9.6; *p* < 0.001; *d* = 1.22, Awake: t(62) = 2.08; *p* = 0.042; *d* = 0.26, Tense: t(62) = 8.88; *p* < 0.001; *d* = 1.13).

### NPU laboratory

**Fig. 2:**
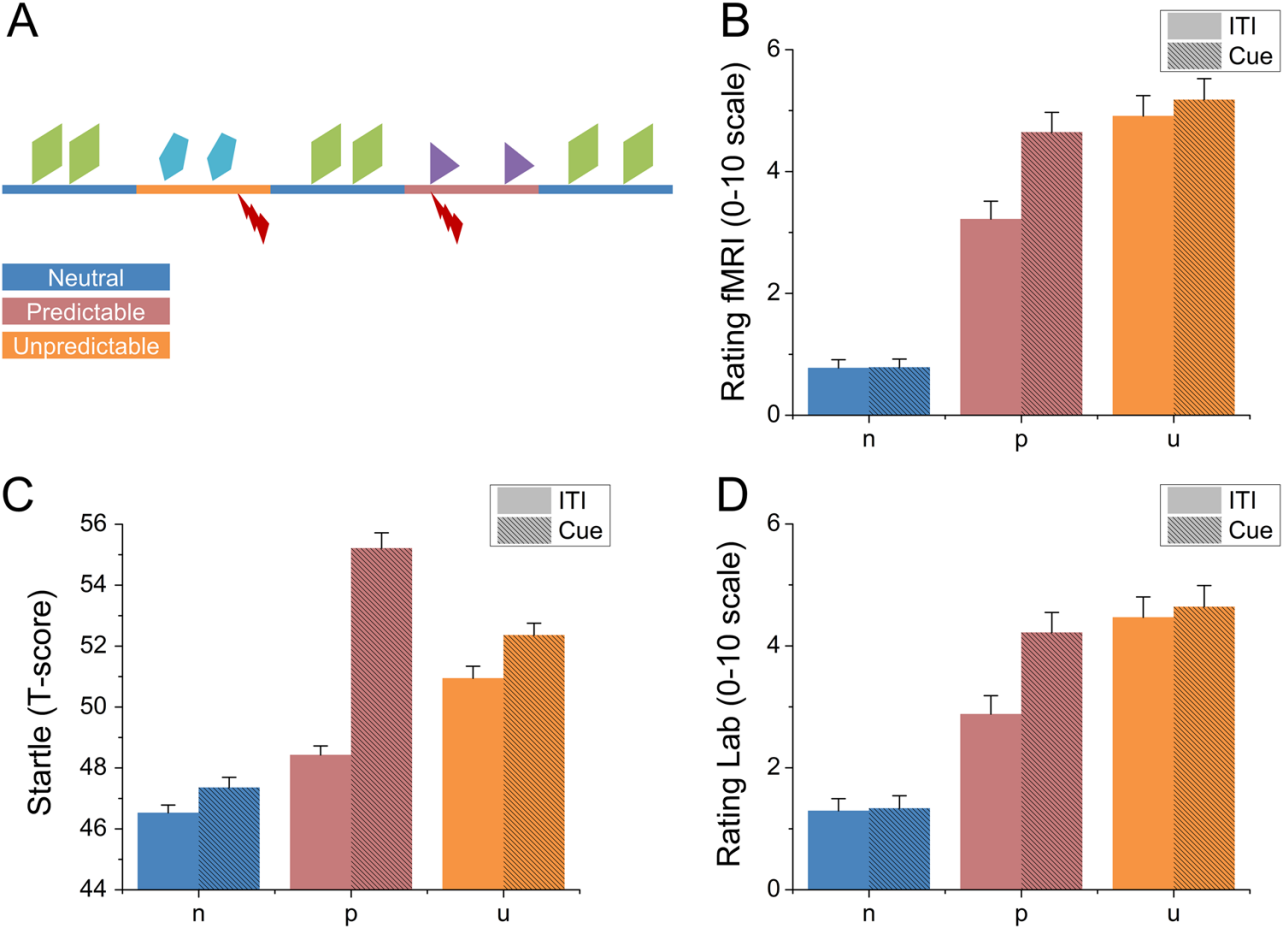
Design and behavioral outcome measures from the NPU sessions **a** Schematic demonstrating design of the NPU paradigm. Colored lines indicate blocks of neutral, predictable, and unpredictable threat. Geometric shapes indicate visual cues presented during blocks. Lightning bolts represent timing of shock delivery. **b** Average anxiety ratings during the fMRI session for the neutral (N), predictable (P), and unpredictable (U) blocks when the cue was present (Cue) or absent (ITI) from the screen. **c** Startle magnitude (*t*-scores) during the laboratory session for the neutral (N), predictable (P), and unpredictable (U) blocks when the cue was present (Cue) or absent (ITI) from the screen. **d** Average anxiety ratings during the laboratory session for the neutral (N), predictable (P), and unpredictable (U) blocks when the cue was present (Cue) or absent (ITI) from the screen. Bars represent the mean ± SEM

To determine the effectiveness of the laboratory session of the NPU threat task, we analyzed the online ratings and startle magnitudes for the N, P, and U blocks using 3 (Block: N, P, U) × 2 (Interval: Cue vs. ITI) repeated-measures ANOVA (See Fig. 2). For both ratings and startle, we found a significant main effect for both block (Ratings: F(2, 124) = 97.99; *p* < 0.001, Startle: F(2, 124) = 73.46; *p* < 0.001) and interval (Ratings: F(1, 62) = 54.14; *p* < 0.001, Startle: F(1, 62) = 95.58; *p* < 0.001), as well as a significant Block × Interval Interaction (Ratings: F(2, 124) = 48.25; *p* < 0.001, Startle: F(2, 124) = 41.81; *p* < 0.001).

To characterize the interaction, we quantified fear and anxiety from the ratings and startle measures. For fear, we subtracted the rating (i.e., FPR) and startle (i.e., FPS) magnitude during the predictable ITI period from the predictable cue period. For anxiety, we subtracted the rating (i.e., APR) and startle (i.e., APS) magnitude during the unpredictable blocks from the neutral blocks during both the cue and ITI. As expected, both ratings and startle increased during the predictable cue compared to the predictable ITI, indicating an acute increase in fear brought on by the predictable cue (FPR: t(62) = 7.36; *p* < 0.001; *d* = 0.93, FPS: t(62) = 10.3; *p* < 0.001; *d* = 1.3). Similarly, both ratings and startle increased during the unpredictable cue (APR-CUE: t(62) = 11; *p* < 0.001, *d* = 1.39; APS-CUE: t(62) = 7.78; *p* < 0.001; *d* = 0.98) and ITI periods (APR-ITI: t(62) = 11.1; *p* < 0.001; *d* = 1.4, APS-ITI: t(62) = 8.37; *p* < 0.001; *d* = 1.05), indicating a sustained increase in anxiety that was present for the entire unpredictable blocks.

### NPU fMRI session

**Fig. 3:**
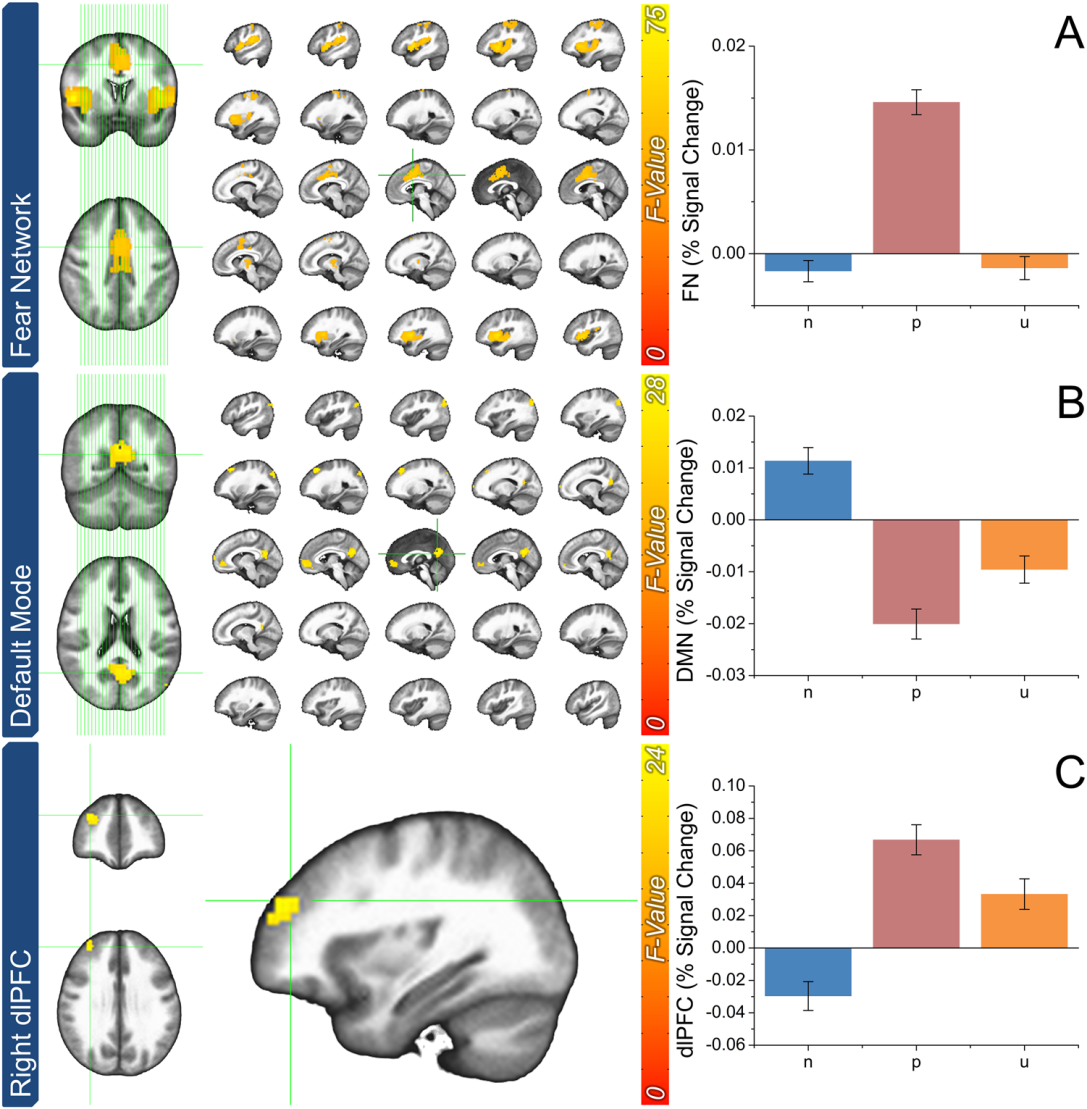
Main effects from the analysis of the BOLD data from the NPU fMRI session **a** Statistical parametric map and corresponding graphic representation for regions of the fear network (FN). **b** Statistical parametric map and corresponding graphic representation for regions of the DMN. **c** Statistical parametric map and corresponding graphic representation for regions of the right dorsolateral prefrontal cortex (dlPFC). Bars represent the mean ± SEM

For the fMRI data, we performed one-way (N, P, U) repeated-measures voxelwise ANOVA on the cue-evoked activity, and extracted the clusters that survived correction for multiple comparisons (see Supplementary Table 1). We then grouped these clusters into two co-activation networks based on their pattern of activity across conditions (see Fig. 3).

Regions in the first co-activation network (FN), including the dmPFC and bilateral insula, showed a pattern of activity consistent with fear only (i.e., P ≠ N and U). Specifically, these regions showed significantly more activity to the predictable than the unpredictable cue (P > U: t(62) = 7.99; *p* < 0.001; *d* = 1) or neutral cue (P > N: t(62) = 11.75; *p* < 0.001; *d* = 1.45).

Regions in the second co-activation network (DMN), including the ventromedial prefrontal cortex and posterior cingulate cortex, showed a pattern of activity consistent with both fear and anxiety (i.e., P and U ≠ N). However, unlike the FN, there was significantly less activity to the predictable cue (P > N: t(62) = −7.89; *p* < 0.001; *d* = −0.97) and the unpredictable cue (U > N: t(62) = −4.96; *p* < 0.001; *d* = −0.62) compared to the neutral cue. Although not reported, the pattern of results for each cluster matches that of the corresponding co-activation network.

The final cluster (Right dlPFC) showed a pattern of activity consistent with both fear and anxiety (i.e., P and U ≠ N), but unlike the regions in the DMN this cluster exhibited significantly more activity to the predictable cue (P > N: t(62) = 7.28; *p* < 0.001; *d* = 0.91) and unpredictable cue (U > N: t(62) = 4.38; *p* < 0.001; *d* = 0.55) compared to the neutral cue.

We did not observe any significant effects in the BNST.

### PCA

**Fig. 4:**
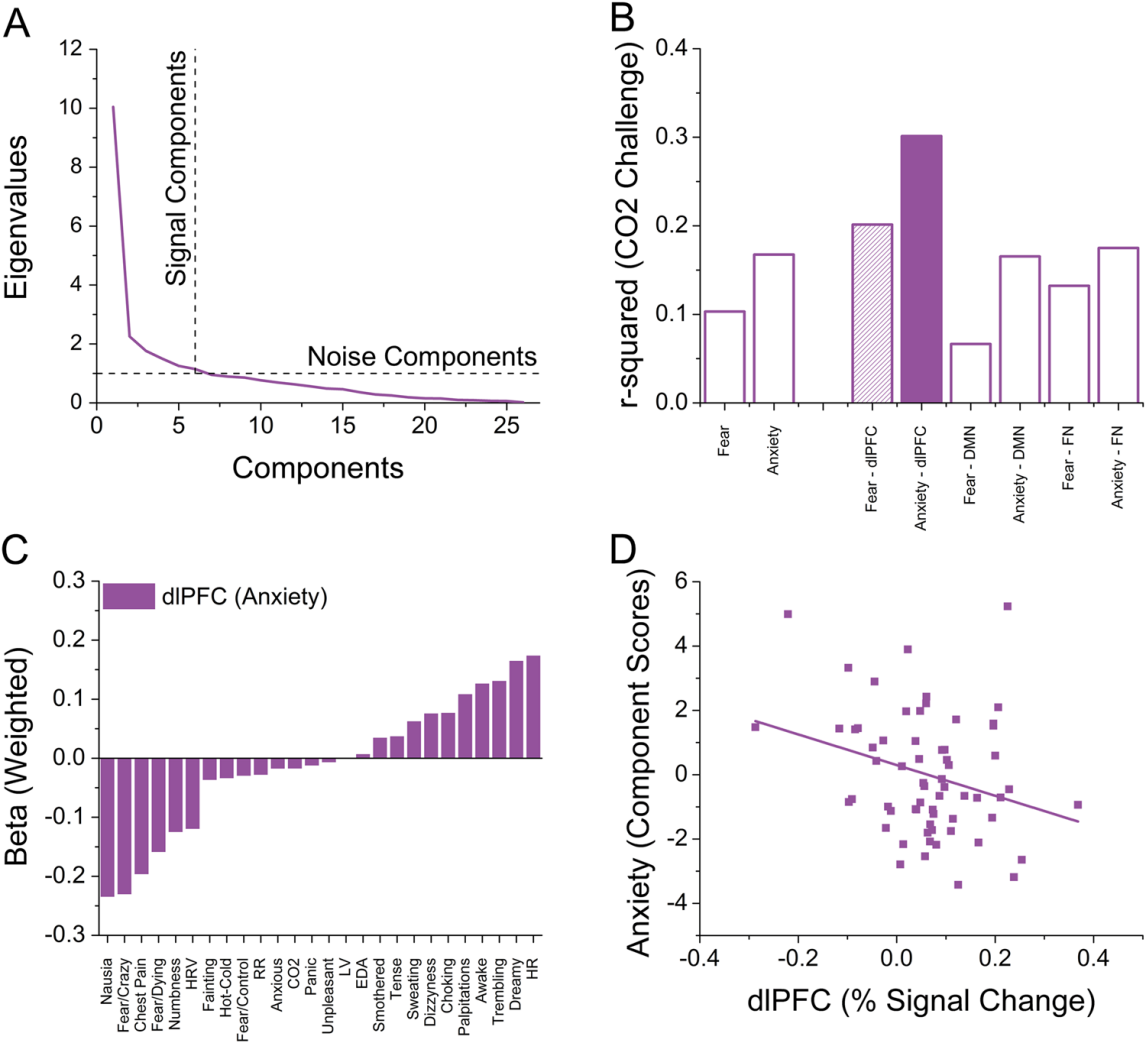
Statistical relationship between measures from the CO_2_ challenge and NPU sessions **a** Screenplot demonstrating outcome of the principal components analyses for the CO_2_ challenge. Components with an eigenvalue >1 are considered signal components, while those with an eigenvalue <1 are considered noise components. **b** Variability (*r* ^2^) in dependent measures from the NPU sessions accounted for by the signal components in the CO_2_ challenge. Filled bars are significant after correcting for multiple comparisons. Hatched bars are trends, but not significant after correcting for multiple comparisons. **c** Weighted beta coefficients showing contributions of specific items from the CO_2_ challenge to the PCA/regression model predicting anxiety-related dlPFC activity. **d** Correlation between anxiety-related dlPFC activity and anxiety (as derived via PCA from NPU startle and ratings during fMRI and laboratory sessions)

To identify regressors of interest, we conducted independent PCAs including all variables from a given experiment. For the CO_2_ experiment, we entered 26 variables into the PCA, and identified six components with an eigenvalue >1 (see Fig. 4a). These six components explained 69.09% of the variability. For the NPU laboratory session, we entered 12 variables into the PCA, and identified three components with an eigenvalue >1 (see Supplementary Fig. 2). These three components explained 69.77% of the variability. For the NPU fMRI session, we entered 33 variables into the PCA, and identified 7 components with an eigenvalue >1 (see Supplementary Fig. 2). These seven components explained 79.25% of the variability.

### GLMs

To determine whether the primary outcome measures of each experiment were affected by individual differences in responding in the other experiments, we conducted a series of GLMs (One per measure, see Supplementary Fig. 3) using the subject scores for the signal components from one experiment to predict the outcome measures from another experiment. This was done in a systematic fashion, whereby the signal components from each experiment were used to predict each outcome measure for the remaining experiments. The subsequent *r* ^2^ values are plotted in Supplementary Fig. 3. Although several of these GLMs were significant at the 0.05 level (see hatched bars in Supplementary Fig. 3), only one GLM (CO_2_ challenge components → Anxiety-related dlPFC activity) was significant after correcting for multiple comparisons (see shaded bar in Fig. 4b).

### Weighted beta coefficients

To characterize the relationship between predictor variables from the CO_2_ challenge and anxiety-related dlPFC activity, we computed weighted coefficients for this GLM (see Fig. 4c). CO_2_ challenge items related to fear or negative affect tended to load negatively on anxiety-related dlPFC activity (f(6, 56) = 4.02; *p* = 0.002; FDR = 0.016; *r* ^2^ = 0.3), suggesting that individuals with high anxiety-related dlPFC activity report less fear during the CO_2_ challenge. A similar but less robust pattern was observed for fear-related dlPFC activity (f(6, 56) = 2.35; *p* = 0.043; FDR = 0.17; *r* ^2^ = 0.2; see Supplementary Fig. 4).

### Anxiety/dlPFC correlations

Because individuals who exhibited larger anxiety-related dlPFC responses during the NPU fMRI session reported less anxiety during the CO_2_ challenge, we hypothesized that these dlPFC responses may regulate anxiety more generally. Thus, we correlated anxiety ratings from the unpredictable condition during both sessions and startle from the NPU Laboratory session with dlPFC responses from the NPU fMRI session. For all measures we calculated Unpredictable > Neutral difference scores. Because the dlPFC responses were cue-evoked, we included separate scores for the cue and ITI periods for the anxiety ratings and startle measures. Importantly, across measures (Ratings vs. Startle), studies (fMRI session vs. Laboratory session), and intervals (Cue vs. ITI), the correlations with anxiety-related dlPFC responses were negative (see Supplementary Table 2). Although many of these correlations were only trends, the pattern is consistent across measures, and these measures themselves were likely correlated. Therefore, we combined the measures using a PCA and extracted a single value component to anxiety (eigenvalue = 3.6). As expected, the anxiety component was significantly negatively correlated with anxiety-related dlPFC activity (r(62) = −0.29; *p* = 0.023; see Fig. 4d). When applying a similar approach to fear and fear-related dlPFC activity, no significant correlations were found (all *p* values > 0.05).

## Discussion

The purpose of this study was to determine the degree to which panic susceptibility is related to (1) fear and anxiety during the NPU threat task and (2) reactivity in the neural systems that mediate fear and anxiety. Accordingly, we exposed individuals to a 7.5% CO_2_ challenge and two versions (laboratory and fMRI) of the NPU threat task. Contrary to our first hypothesis, we did not find evidence that panic symptoms were mediated by the same core neural system as anxiety. However, we did find evidence that panic symptoms to CO_2_ may be regulated by the same neural system that regulates anxiety to unpredictable threat. When comparing across experimental sessions, we found that CO_2_-related behavioral changes were associated only with dlPFC activity during the unpredictable cue (see Fig. 4b). The activity of the dlPFC is commonly considered to mediate cognitive control^51,52^, and our results suggest that the dlPFC regulates anxiety in these paradigms^48^. Weighted coefficients from our analysis showed that fear-related DSQ symptom items negatively loaded onto dlPFC activity (see Fig. 4c). Similarly, anxiety-related dlPFC was negatively correlated with anxiety during the NPU sessions (see Fig. 4d). Together, these results suggest that panic symptoms and anxiety are regulated by the same prefrontal cognitive control system^48^.

In addition to this novel finding, we replicate many previous reports about the CO_2_ challenge and the NPU threat task. During the CO_2_ challenge, subjects showed increased respiration, physiological arousal, and self-reported anxiety (see Fig. 1). During the laboratory NPU session, subjects showed the traditional pattern of elevated startle and self-reported anxiety during the predictable cue (fear), and unpredictable cue and ITI periods (anxiety)^8,15,53–55^ (see Fig. 2). During the NPU fMRI session, subjects showed distinct patterns of BOLD activity related to fear and anxiety^22,56–61^ (see Fig. 3). During both predictable and unpredictable cues, subjects showed decreased DMN activity (see Fig. 3b), and increased dlPFC activity (see Fig. 3c). However, only the predictable cue increased FN activity (see Fig. 3a). This FN result is consistent with the fact that only the predictable cue informed the probability of shock^15^.

Previous research has shown that panic attacks are not necessarily mediated by the same mechanisms as fear^14,62^. However, there is evidence that history of panic attacks is related to startle magnitude during unpredictable threat^10–12^. There are two possible explanations for this observation. First, panic susceptibility may be driven by elevated responding in anxiety-related neural systems^22,56–61^. Alternatively, panic susceptibility may be driven by attenuated responding in anxiety-regulating neural systems^63–65^. We found that panic symptoms can predict dlPFC activity, which is negatively associated with anxiety, which supports the second hypothesis, and suggests that the dlPFC regulates panic symptoms and anxiety. Consistent with this, anxiety patients show dlPFC deficits during complex working memory (WM) tasks^27^. In contrast, we found no evidence that panic symptoms are associated with anxiety-related regions, which fails to support the expression hypothesis (see Fig. 4b). Thus, more work is needed to understand the distinct neural systems mediating the expression of anxiety and panic.

These findings can explain the previously counterintuitive finding that CO_2_ administration actually reduces startle magnitude^66–68^. According to our results, one could argue that the dlPFC is a common regulatory system engaged by 7.5% CO_2_ and by unpredictable threat because these challenges evoke a similar defensive response. Thus, evoking either defensive response should lead to the regulation of both responses. According to this hypothesis, startle is reduced during CO_2_ administration because 7.5% CO_2_ administration engages the dlPFC, which regulates the ongoing activity in the neural system engaged by unpredictable threat. Therefore, it should also be possible to reduce CO_2_-related panic symptoms using sustained unpredictable threat, as a proof of concept. This hypothesis is testable within the context of the rapid 35% CO_2_ challenge. In this paradigm, subjects inhale a single breath of air with 35% CO_2_, which results in panic attacks in susceptible individuals^69–71^. Therefore, using unpredictable threat to engage the system that mediates anxiety should reduce the likelihood of experiencing a panic attack in this paradigm.

It may also be possible to test the regulation hypothesis using other techniques to drive dlPFC activity^72–82^. We have shown previously that WM, known to activate the dlPFC, is sufficient to reduce APS during threat^83,84^. We know of no studies examining the effect of WM on panic symptoms. Future studies might accomplish this by administering single breaths of 35% CO_2_ during periods of low vs. high WM load^69–71^. According to the regulation hypothesis, subjects should experience fewer panic attacks during high WM blocks compared to low WM blocks. The N-back WM task would be ideal because it provides long durations of steady-state WM engagement, where WM load can be parametrically manipulated within subjects^79–82^. It may also be possible to test the regulation hypothesis using noninvasive neuromodulation. For instance, one could administer single breaths of 35% CO_2_ while the subject receives electrical (transcranial direct current stimulation; tDCS^72–74^) or magnetic (transcranial magnetic stimulation; TMS^75–78^) to the right dlPFC. According to the regulatory hypothesis, subjects should experience fewer panic attacks during excitatory (i.e., anodal tDCS or high-frequency TMS) compared to inhibitory (i.e., cathodal tDCS or low-frequency TMS) or sham stimulation of the dlPFC.

### Strengths and limitations

There were a number of strengths and limitations with current work that should be noted. Among the primary strengths, we used well-validated, experimental, translational techniques for inducing symptoms of fear (predictable shock threat), anxiety (unpredictable shock threat), and panic (7.5% CO_2_ inhalation)^4,15^, and included an adequate sample size (*n* = 63) to test the hypotheses. Among the primary limitations, we did not directly assay neural activity during the CO_2_ challenge, and we did not report the findings related to sustained anxiety from the unpredictable blocks in the fMRI study. For a full discussion of these strengths and limitations, see the Supplementary Materials.

## Conclusions

The purpose of this study was to determine the degree to which panic symptoms correlated with fear, anxiety, and the neural systems that mediate/regulate fear and anxiety. We found evidence that panic symptoms and anxiety may be regulated by similar prefrontal cognitive control mechanisms. Accordingly, these results raise several testable hypotheses about the effect of cognitive control on panic symptoms, and warrant future studies on this topic.

## Electronic supplementary material

Supplemental Material
