# Supplementary material for: The relationship between dlPFC activity during unpredictable threat and CO2-induced panic symptoms
Source: Transl Psychiatry. 2017 Nov 30;7:1266. doi: 10.1038/s41398-017-0006-5 (PMC5802456; doi:10.1038/s41398-017-0006-5)
Supplement: Supplementary file 1 — Supplemental Material [file 41398_2017_6_MOESM1_ESM.docx]

# Supplemental Methods

**CO2 challenge: Panic-like Symptoms.** The Diagnostic Symptom Questionnaire (DSQ) is a 16-item self-report measure that assesses the symptoms of a panic attack as outlined by the Diagnostic and Statistical Manual of Mental Disorders’ (DSM-5) ^1^. The first 12 items assess the presence and level of discomfort of somatic symptoms (e.g. “Trembling or shaking”, “Chest tightness or pain”, “Choking”) and the next 4 items assess the presence and intensity of cognitive symptoms (e.g. “Fear of dying”, “Fear of going crazy”, “Fear of losing control”, “Feeling scared or panicky”), both using an 8-point Likert scale (e.g. 0 = “Not at all noticed”, 4 = “Moderately felt”, 9 = “Very strongly felt”). The DSQ was administered four times: 1) during the No-Facemask Pre-experiment Period, 2) at the end of the Pre-CO2 period, 3) three minutes after the start of the CO2-Inhalation period, and 4) immediately after the Recovery period (while still wearing the facemask).

The Subjective Units of Distress Scale (SUDS) ^2^ is a 4-item self-report measure that assesses subjective anxiety and vigilance using a 1-10 point semantic scale (e.g. 1 = “Not anxious at all”, 10 = “Extremely anxious”, 1 = “Sleepy”, 10 = “Awake”). SUDS was administered every two minutes during the 18-minute CO2 challenge task, starting at two minutes into the Room Air Breathing Period and ending at five minutes after the start of the Recovery Period, for a total of nine SUDS ratings during the challenge task. In addition, SUDS was administered during the No-Facemask Pre-experiment Period.

**CO2 challenge: Psychophysiological Symptoms**. Tidal Volume (LV) and capnography (CO2%) were continuously measured during room-air and CO2 air exposure using breath-by-breath spirometry. Heart rate (HR), heart rate variability (HRV), skin conductance (SC), and respiratory rate (RR) were continuously measured as indices of sympathetic arousal throughout the CO2 challenge task. HR was collected using a two-lead electrocardiogram with lead placement on the left brachial and right axillary arteries. SC was collected with the placement of two disposable electrodes on the base of the proximal phalanges of the first two digits and the *abductor digiti minimi* of the non-dominant hand. RR was collected with the placement of a respiratory transducer around the upper abdomen, and all data were sampled at a rate of 2000 Hz. A Biopac data acquisition unit (MP150; BiopacSystems Inc., US) with Acknowledge 4.4 software was used to acquire, clean, filter, and analyze the psychophysiological data.

**NPU Laboratory Session: Acoustic Startle Stimulus.** The startle stimulus was a 40-ms burst of a 103 dB white noise (near instantaneous rise/fall times) delivered via the computer soundcard to standard over-the-ear headphones. Prior to the experiment, the subject underwent a habituation block with 9 un-signaled presentations of the white noise burst.

**NPU Laboratory Session: Physiological Responses.** Eye-blink startle responses to acoustic startle stimuli were measured with electromyography activity in the orbicularis oculi muscle using two tin cup electrodes placed under the left-eye. EMG was sampled at a rate of 2000 Hz, rectified, filtered, and integrated using a Biopac data acquisition unit (MP150; BiopacSystems Inc., US) with Acknowledge 4.4 software.

**NPU Sessions: Online Anxiety Ratings.** Subjective anxiety was measured continuously with an online Likert-style number dial (e.g. 0 = “Not anxious at all, 10 = “Extremely anxious”) displayed in the center of the screen. Participants received explicit instructions to update the rating with keyboard presses on a moment-to-moment basis following any changes in anxiety.

**NPU Sessions: Shock.** The shock consisted of a 100 ms, 200 Hz train of stimulations delivered to the left wrist via 11mm disposable Ag/AgCl electrodes, using a constant current stimulator (DS7A, Digitimer, LLC, Ft. Lauderdale, FL). The intensity of the shock was set prior to the experiment using a workup procedure. During this procedure, subject received a series of stimulations of increasing intensity until they rated the stimulation as “uncomfortable but not painful”. This intensity was used for the remainder of the session.

**NPU fMRI Session: MRI acquisition.** We collected 2 runs, each containing 200 multi-echo EPI images, using a 3T General Electric Signa HDxt (Waukesha, WI) fMRI system, and an 8-channel head coil. For each whole brain image, we collected 30 interleaved 3.5 mm slices (matrix = 64 × 64; FOV=224 mm × 224 mm) parallel to the AC-PC line (TR=2.5s; TEs = 18.4 ms, 39.5 ms, 60.6 ms; flip angle = 75°). We also acquired a T1-weighted MPRAGE (TR = 7796 ms; TEs = 2.98 ms, 7.27 ms; flip angle = 7°) with 172, 1 mm axial slices (matrix = 256 mm × 256 mm; field of view (FOV) = 256 mm × 256 mm) for registration with the EPI images.

**NPU fMRI Session: MRI processing.** The AFNI software package was used to preprocess and analyze the MR images ^3^. EPI volumes for each of the 3 echoes were first preprocessed using standard techniques. The first 4 EPI volumes were discarded to allow the magnetic field to reach equilibrium. Then the images were despiked, slice-time corrected, deobliqued, volume-registered, and skull-stripped.

The preprocessed EPI volumes were then denoised using the echo time (TE) dependent independent components analysis for multi-echo fMRI (multi-echo ICA) ^4^. This technique removes sources of noise corresponding to non-BOLD-like artifacts, such as motion, physiology, and scanner artifact ^4^. In brief, this process decomposes the timeseries into independent components using FastICA, then determines the degree to which each of the components decays across the echoes. Components that do not decay in a TE-dependent pattern, consistent with the T2* decay of BOLD signals, are discarded, and a new denoised timeseries is synthesized from the remaining components.

The denoised timeseries for each run were then co-registered and scaled, and entered into a general linear model (GLM) to identify BOLD responses to the cues in the N, P, and U periods. Cues were modeled using an 8 s block convolved with the hemodynamic response function (HRF). The baseline was estimated using a series of regressors of no interest including the six motion parameters from the volume registration step, 0-4^th^ order polynomials to model baseline drift, and HRFs for any event of no interest (i.e. shocks and button presses). Finally, the timeseries were “scrubbed” for motion using the derivative of the motion regressors from the volume registration step. Those images with a Euclidean norm above 0.5 mm were censored. Any subject with more than 10% of their volumes censored were excluded from the analysis.

The T1-MPRAGE images were used to normalize the EPI images to MNI space, and to mask out non-grey matter voxels. First the T1 images were processed with Freesurfer using the standard pipeline ^5,6^. Next the skull-stripped T1 images were normalized to the MNI_avg152T1 template distributed with AFNI, using non-linear registration (3dQwarp). Single subject grey matter maps were created using all grey matter atlas regions from the Freesurfer output, downsampled to the EPI resolution, and dilated by 1 voxel. A group-level grey matter mask was created by combining single-subject grey matter masks, and thresholding at 2/3 overlap ^7^. Next the original T1 images were aligned to the EPI images using a non-linear criterion based on the local Pearson correlation ^8^. The inverse of this transformation matrix, plus the T1-to-MNI transformation matrix were used to normalize the EPI images to MNI space. Then the group-level grey matter mask was used to mask out non-grey matter voxels, and the remaining voxels were blurred using a 6mm FWHM Gaussian kernel. Normalized/blurred beta images corresponding to the cues in the N, P, and U conditions were used in all subsequent analyses.

## Data analysis

**CO2 challenge.** Raw breath was first bandpass filtered (0.05 Hz – 1 Hz), and then thresholded to identify the peak-to-peak RR for the Pre-CO2, CO2-Administration, and Recovery periods. To identify changes in LV, breaths were identified using upper and lower thresholds in the air flow channel. The magnitude of these breaths was averaged within each phase of the challenge (Pre-CO2, CO2-administration, and Recovery). Similarly, changes in CO2 exhalation were assessed by identifying breaths in the capnography channel, and averaging the magnitude of CO2 changes for the breaths in each phase of the challenge. EKG was first bandpass filtered, (0.5 Hz – 1 Hz) and then thresholded to identify the R-peak-to-R-peak rate for each phase of the challenge. HRV was calculated automatically by the software by identifying the variability in the R-peak-to-R-peak rate. EDA was averaged within each phase of the challenge. Responses to the DSQ and SUDS items were averaged across repetitions within each phase of the challenge.

For each measure, we created difference scores by subtracting values during the Pre-CO2 period from values during the CO2 administration period for variables reflecting: breathing (RR, LV, end-tidal CO2), physiological arousal (HR, HRV, EDA), subjective panic symptoms (DSQ items), subjective emotional state (SUDS: Unpleasant, Anxious, Awake, Tense). These values were then compared to 0 using a t-test to determine the effectiveness of the CO2 challenge.

**NPU laboratory.** Startle responses were analyzed according to previous recommendations ^9^. Data were bandpass filtered (30 Hz – 300 Hz), smoothed (20 ms sliding-window), and rectified. Afterward, the peak (20 ms – 100 ms after the startle probe) minus the baseline (mean during 50 ms prior to the startle probe) values were extracted from the EMG channel for each startle probe. These values were then normalized and converted to T-scores (t = z * 10 + 50). Anxiety ratings were recorded continuously, and the values were extracted at the onset of each startle probe. Startle magnitudes and anxiety ratings were analyzed using a 3 (Block: N,P,U) x 2 (Interval: Cue vs. ITI) repeated-measures ANOVA.

**NPU fMRI.** Using the masked and blurred beta maps, we first conducted 1-way repeated measures ANOVA to identify significant differences in activity evoked by the cues in the N, P, and U blocks. We then thresholded this parametric map, and extracted the voxels showing a significant main effect of cue type. The size, XYZ coordinates, and effect size for each cluster is plotted in Table 1. To characterize the main effect, we followed up this analysis with a series of paired pairwise comparisons on the average voxel intensity for each individual cluster, using standard statistical software. These pairwise comparisons were used to group clusters according to two criteria: 1) whether they showed a pattern consistent with fear (P ≠ N & U), anxiety (U ≠ N & P), or both fear and anxiety (P & U ≠ N), and 2) whether the overall effect was positive (i.e. P and/or U > N) or negative (P and/or U < N). Cohen’s d for each of these comparisons is plotted for each cluster in Table 1. Paired-sample t-tests corresponding to these comparisons were then computed for each cluster group (co-activation network).

We corrected for multiple comparisons across voxels using a cluster-based approach ^10^. We began by estimating the smoothness of our error using the residual timeseries from the single-subject first level analyses. We used a Gaussian plus mono-exponential shaped function, as implemented by the “-acf” flag in 3dFWHMx. We then averaged the parameter estimates from this model across subjects, and generated 10,000 random parametric maps of the same smoothness using 3dClustSim. Next, we identified the largest cluster for each simulation at a voxel-wise alpha of 0.001, and identified a minimum cluster size threshold based on this distribution. As a result, we thresholded our data using a voxel-wise p-value of 0.001, and a minimum cluster size of 40 contiguous voxels, which corresponded to a 2-tailed alpha of 0.05.

**Correlations.** In order to understand the relationship between the neural and behavioral responses in these paradigms, we first examined the correlations among the dependent measures recorded in each experiment. For the CO2 challenge, we included each question of the DSQ, the psychological state questions (Unpleasant, Anxious, Awake, Tense), and the physiological recordings (RR, LV, end-tidal CO2, HR, HRV, EDA). Because we were specifically interested in the effect of the CO2 challenge, rather than the raw values for each of these measures, we used the [CO2 administration – Pre-CO2] difference scores. For the NPU laboratory session, we used the ratings and startle magnitudes for the cue and ITI periods of the neutral, predictable, and unpredictable blocks. For the fMRI data, we used the cue-evoked activity for the neutral, predictable, and unpredictable blocks for each of the 11 functionally-defined ROIs reported in Table 1.

We used permutation testing to correct for multiple comparisons. On each of the 10,000 permutation tests, each column (i.e. independent variable) was shuffled randomly with respect to every other column, the cross-correlation matrix was computed, and the correlation with the largest absolute value was recorded. We used the distribution of these values to identify a threshold for the correlation coefficients in this table. We chose the value in this distribution that was at the 97.5^th^ percentile (alpha/2).

**PCA.** Because the previous correlation matrix included a large number of variables, correcting for multiple comparisons may have resulted in Type II error. To rule out this possibility, we used a data reduction strategy that combined principal components analyses (PCAs) and general linear models (GLMs). First, we used independent PCAs for each experiment to reduce the number of factors in the predictive models. Each PCA included all variables from a given experiment that appeared in the global correlation matrix. Components with an eigenvalue > 1 (Kaiser method) ^11^ were marked as signal components, while those with an eigenvalue < 1 were marked as noise components. The scores for each subject and each component were used as predictors in the subsequent GLMs.

**GLMs.** Next, we used a round robin technique to determine how variability in a given experiment affects various responses in the other experiments. First we narrowed down our outcome measures to those that reflected the effect of interest for each experiment. For the CO2 challenge experiment, we used the [CO2 administration – Pre-CO2] difference scores. We averaged across DSQ questions to get a sum for the DSQ, and averaged across the psychological questionnaire to get a value reflecting negative affect, but included the physiological recordings as separate outcome measures. For the NPU laboratory session, we averaged the FPS and FPR scores to get a measure reflecting Fear to the predictable cue, and averaged the APS and APR scores to get a measure reflecting anxiety during the unpredictable blocks. For the NPU fMRI session, we created fear and anxiety difference scores by subtracting the neutral cue evoked BOLD activity from the predictable (Fear) and unpredictable (Anxiety) cue evoked BOLD activity for the FN, DMN, and dlPFC (See Results section). It should be noted that we included only functionally defined regions of interest in the analysis (See NPU fMRI section).

Once we identified a reduced list of outcome measures for each experiment, we then used GLMs from the remaining two experiments to predict these outcome measures. First we ran a model with all noise components as regressors of no interest, and retained the residuals from this model. Next we ran a model with the signal components from a given experiment as regressors of interest, and extracted the r^2^ value. Importantly, because we only used components with an eigenvalue > 1 in this model, and because these components are orthogonal, the r^2^ value of this model represents the maximum variability in the outcome measure that can be accounted for by the variability in measures from the predictor experiment. We completed this process for each outcome measure and each (predictor) experiment, and used FDR adjustment to correct for multiple comparisons.

**Weighted beta coefficients.** To characterize the relationship between individual predictor variables and the outcome measures, we computed weighted beta values by multiplying the beta coefficients for each signal component entered into a specific GLM by the individual item loadings for the corresponding signal component. We then summed theses weighted betas across components. The result is a single number reflecting the degree to which a given predictor variable in a given experiment contributes to the ability of that experiment to predict the outcome measure. For instance, we identified 6 signal components for the CO2 challenge, and used these to predict anxiety-related dlPFC activity. To understand how a given item from the CO2 challenge contributed to the prediction of anxiety-related dlPFC activity, we looked at how that item contributed to each signal component, weighted by how much that signal component contributed to the overall prediction.

# Supplemental Results

## NPU fMRI session: behavior

To determine the effectiveness of the fMRI session of the NPU threat task, we analyzed the online ratings for the N, P, and U blocks using 3 (N,P,U) x 2 (Cue vs. ITI) repeated-measures ANOVA (See Figure 2). As with the ratings in the laboratory session, we found a significant main effect for both Block (F(2, 124) = 161.16; p < 0.001) and Interval (F(1, 62) = 40.31; p < 0.001), as well as a significant Block x Interval Interaction (F(2, 124) = 28.87; p < 0.001). As with the laboratory session, we created scores for fear and anxiety using the online ratings. Consistent with the laboratory session, we found significant evidence for the expression of both fear (FPR: t(62) = 5.75; p < 0.001; d = 0.72) and anxiety (APR: t(62) = 13.42; p < 0.001; d = 1.69).

## Correlations

In order to understand the relationship between the neural and behavioral responses in these paradigms, we first examined the correlations among the dependent measures recorded in each experiment (See Supplemental Table 1). We began by computing the cross-correlation matrix for all independent variables listed in the methods section for all 3 experiments (See Supplemental Figure 1). We used permutation testing to identify a threshold for the correlation coefficients (t = 0.57). After thresholding the correlation matrix, we found that although there were robust within-experiment correlations, there were no between-experiment correlations that survived correction for multiple comparisons.

## Weighted beta coeficients

In addition to the findings with the CO2 challenge data, there were several trends with the NPU laboratory session data and the NPU fMRI session data. For instance, NPU laboratory session data was able to predict both negative affect (SUDS: f(3, 59) = 2.9; p = 0.042; FDR = 0.27; r^2^ = 0.13; See Supplemental Figure 4) and RR (f(3, 59) = 4.55; p = 0.006; FDR = 0.086; r^2^ = 0.19; See Supplemental Figure 4), with anxiety ratings during the unpredictable threat condition loading positively onto both outcome measures (i.e., greater anxiety lead to greater RR). Finally, data from the NPU fMRI session predicted fear during the NPU laboratory session (f(7, 55) = 2.45; p = 0.029; FDR = 0.264; r^2^ = 0.24; See Supplemental Figure 4). This is not surprising, considering a large proportion of the NPU fMRI findings were localized to the fear network and specific to the predictable cue. Indeed when the weighted betas are plotted, it is the predicted-cue-related activity in the fear network that loads most highly onto fear in the NPU laboratory session.

# Supplemental Discussion

Consistent with the anxiety-related dlPFC findings, we were also able to use the CO2 challenge symptoms to predict fear-related dlPFC activity (albeit at a trend level). However unlike the anxiety findings, we were unable to detect a regulatory relationship between fear and fear-related dlPFC activity. This is the case even though the effect size for fear-related dlPFC activity is nominally larger than that for anxiety-related dlPFC activity. Together these results suggest that the dlPFC does not solely regulate negative affect. Instead, we hypothesize that the dlPFC regulation of negative affect is a byproduct of the general attentional control function of this region ^12,13^. In the case of the NPU paradigm, the cue during the unpredictable condition does not yield any useful information about the shock ^14^. Therefore, this stimulus could be considered a distractor. Accordingly, the degree to which this cue activates the dlPFC may index capacity for distractor suppression ^15,16^. In contrast, the cue during the predictable condition contain useful information about the shock. Therefore, this stimulus could be considered less of a distractor, and the dlPFC activity evoked by this cue may serve other purposes. It has been shown that the prefrontal cortex plays a key role in the timing of events during fear conditioning ^17,18^, which may be the case here.

In addition to the data in the manuscript, there were several other trends when comparing across experimental sessions. For instance, data from the NPU laboratory session was able to predict (at trend levels) both the SUDS scores and the respiratory rate during the NPU session, and this relationship was driven primarily by the ratings in the NPU laboratory session. At least for the SUDS, which also represents subjective arousal ^2^, the relationship between subjective anxiety ratings makes sense. However, for RR, the relationship is less clear, and may suggest that increases in RR are driven by subjective affective state. In other words, panic is marked by increased respiratory symptoms, and individuals anxious about panic attacks may increase their respiratory rate to alleviate these symptoms ^19^. In addition, fMRI data during NPU was able to predict (at a trend level) fear during the NPU laboratory session. Importantly, the strongest positive predictor of fear in the NPU laboratory session was activation of regions of the fear network during the predictable cue ^17,20,21^. This not only replicates previous work with fear conditioning ^22,23^, but it serves as a manipulation check for the combined PCA/Regression approach used in the current work. Although the findings in this section support the main finding of the current work, they are only trends, and should be approached with caution. Future work should be conducted to explore these relationships.

## Strengths and limitations

There are a number of strengths with the current work that should be noted. First, this study was conducted with strong hypotheses based on prior data linking panic to responses to unpredictable threat ^24,25^. Second, we included an adequate sample size (63 subjects) with clean data from all three experiments. Third, we used well-established, experimental, translational techniques for inducing symptoms of fear (predictable shock threat), anxiety (unpredictable shock threat), and panic (7.5% CO2 inhalation) ^26,14^. Fourth, we included a comprehensive battery of psychological, psychophysiological, and neural measures to gauge the impact of these emotional manipulations across subjects. Finally, we used a robust statistical modelling procedure that allowed us to capture the maximal amount of shared variance across experiments, while minimizing and correcting for multiple comparisons.

The study also had limitations. First, although we were interested in the patterns of neural activity that maintain and regulate panic symptoms, we were only able to explore this relationship indirectly using the current approach. This is because changes in CO2 levels in the blood fundamentally alter blood oxygenation, thereby compromising our primary dependent measure (BOLD) ^27–29^. Future studies should be conducted using techniques that do not rely on the BOLD response (e.g., magnetoencephalography, etc.).

Second, we did not discuss sustained activity during the unpredictable condition. Unlike fear, anxiety is a sustained state of elevated arousal, and this response may have been better modelled using a block design. However, we chose to model the cue-evoked response for two reasons. First, it allows a statistically symmetric comparison between all three conditions (neutral, predictable, and unpredictable). Second, in the laboratory randomly timed white noise presentations serve as random probes of ongoing activity. Given that the cues share this characteristic (randomly timed onset), we thought this would be a good parallel with the NPU laboratory session.

Third, as mentioned above, we presented randomly timed white noise presentations during the laboratory session, which we used to assess the physiological effects of the predictable and unpredictable threats. Unfortunately, because of technological limitations we were unable to deliver these white noise presentations in the MRI scanner during the fMRI version of the task. Because of this, we were unable to compare the physiological effects of the predictable and unpredictable threats across the separate environments. To overcome this limitation, we implemented a continuous rating scale that subjects used throughout the task in both laboratory and fMRI versions of the NPU task. As can be seen from Figure 2, subjects show a comparable pattern of online ratings in the laboratory and fMRI versions of the task, suggesting that the experience of the subjects was similar across both sessions.

Fourth, our study did not replicate the findings that unpredictable shock threat activates the BNST ^30,31^. One possible reason is that our cue-evoked analysis was not sensitive to the tonic response of the BNST. To determine whether this was the case, we reanalyzed the data using a block design. Although we observed block-evoked activity for the unpredictable shock condition in the insula and dACC (not shown), we did not observe such activity in the BNST. The more likely explanation is that the BNST is a small structure, difficult to image with 3T ^7^.

Finally, the current work is limited by the use of the combined PCA/regression approach. A more direct/exploratory approach would be to conduct all possible pairwise correlations between dependent measures in the three experiments. However, even using non-parametric permutation tests to correct for multiple comparisons, it was not possible to detect any significant cross-experiment relationships. This is because of the large number of possible correlations (i.e. correlations > total N), resulting in likely Type II errors. To correct for this, we used principal component analyses to reduce the number of predictor variables. The strength of this approach is that it captures much of the variability of the original data, while substantially reducing the number of comparisons. The limitation of this approach is that it is often difficult to interpret the resulting components. Therefore, although this approach allows us to link CO2 challenge symptoms to anxiety-related dlPFC activity, the interpretation of this link (i.e. the evaluation of the weighted beta coefficients) is qualitative. However, we feel that this novel qualitative relationship is sufficiently interesting to warrant further investigation, and suggests that future work should explore the relationship between dlPFC activity and panic susceptibility.

# Supplemental References

1 American Psychiatric Association. *Diagnostic and Statistical Manual of Mental Disorders: Dsm-5*. Amer Psychiatric Pub Incorporated: Arlington, VA, 2013.

2 Wolpe J. The practice of behavior therapy. Pract. Behav. Ther. 1973.

3 Cox RW. AFNI: software for analysis and visualization of functional magnetic resonance neuroimages. *Comput Biomed Res* 1996; **29**: 162–73.

4 Kundu P, Inati SJ, Evans JW, Luh WM, Bandettini PA. Differentiating BOLD and non-BOLD signals in fMRI time series using multi-echo EPI. *Neuroimage* 2012; **60**: 1759–1770.

5 Desikan RS, Ségonne F, Fischl B, Quinn BT, Dickerson BC, Blacker D *et al.* An automated labeling system for subdividing the human cerebral cortex on MRI scans into gyral based regions of interest. *Neuroimage* 2006; **31**: 968–80.

6 Fischl B, van der Kouwe A, Destrieux C, Halgren E, Ségonne F, Salat DH *et al.* Automatically parcellating the human cerebral cortex. *Cereb Cortex* 2004; **14**: 11–22.

7 Torrisi S, O’Connell K, Davis A, Reynolds R, Balderston NL, Fudge JL *et al.* Resting state connectivity of the bed nucleus of the stria terminalis at ultra-high field. *Hum Brain Mapp* 2015; **36**: 4076–4088.

8 Saad ZS, Glen DR, Chen G, Beauchamp MS, Desai R, Cox RW. A new method for improving functional-to-structural MRI alignment using local Pearson correlation. *Neuroimage* 2009; **44**: 839–848.

9 Blumenthal TD, Cuthbert BN, Filion DL, Hackley S, Lipp O V., Van Boxtel A. Committee report: Guidelines for human startle eyeblink electromyographic studies. *Psychophysiology* 2005; **42**: 1–15.

10 Forman SD, Cohen JD, Fitzgerald M, Eddy WF, Mintun MA, Noll DC. Improved assessment of significant activation in functional magnetic resonance imaging (fMRI): use of a cluster-size threshold. *Magn Reson Med* 1995; **33**: 636–647.

11 Jolliffe IT. *Principal Component Analysis*. 2002 doi:10.1007/b98835.

12 Cieslik EC, Zilles K, Caspers S, Roski C, Kellermann TS, Jakobs O *et al.* Is there one DLPFC in cognitive action control? Evidence for heterogeneity from Co-activation-based parcellation. *Cereb Cortex* 2013; **23**: 2677–2689.

13 Rogasch NC, Daskalakis ZJ, Fitzgerald PB. Cortical inhibition of distinct mechanisms in the dorsolateral prefrontal cortex is related to working memory performance: A TMS-EEG study. *Cortex* 2015; **64**: 68–77.

14 Schmitz A, Grillon C. Assessing fear and anxiety in humans using the threat of predictable and unpredictable aversive events (the NPU-threat test). *Nat Protoc* 2012; **7**: 527–32.

15 Levy BJ, Anderson MC. Purging of memories from conscious awareness tracked in the human brain. *J Neurosci* 2012; **32**: 16785–94.

16 Koechlin E, Ody C, Kouneiher F. The architecture of cognitive control in the human prefrontal cortex. *Science* 2003; **302**: 1181–1185.

17 Knight DC, Cheng DT, Smith CN, Stein EA, Helmstetter FJ. Neural substrates mediating human delay and trace fear conditioning. *J Neurosci* 2004; **24**: 218–28.

18 Gilmartin MR, Balderston NL, Helmstetter FJ. Prefrontal cortical regulation of fear learning. *Trends Neurosci* 2014; **37**: 445–464.

19 Rapee RM, Sanderson WC, McCauley PA, Di Nardo PA. Differences in reported symptom profile between panic disorder and other DSM-III-R anxiety disorders. *Behav Res Ther* 1992; **30**: 45–52.

20 Fullana MA, Harrison BJ, Soriano-Mas C, Vervliet B, Cardoner N, Àvila-Parcet A *et al.* Neural signatures of human fear conditioning: an updated and extended meta-analysis of fMRI studies. *Mol Psychiatry* 2015; **21**: 500–508.

21 Knight DC, Smith CN, Stein EA, Helmstetter FJ. Functional MRI of human Pavlovian fear conditioning: patterns of activation as a function of learning. *Neuroreport* 1999; **10**: 3665–3670.

22 Schultz DH, Balderston NL, Helmstetter FJ. Resting-state connectivity of the amygdala is altered following Pavlovian fear conditioning. *Front Hum Neurosci* 2012; **6**: 1–10.

23 Sehlmeyer C, Schöning S, Zwitserlood P, Pfleiderer B, Kircher T, Arolt V *et al.* Human fear conditioning and extinction in neuroimaging: a systematic review. *PLoS One* 2009; **4**: e5865.

24 Grillon C, Ameli R, Goddard A, Woods SW, Davis M. Baseline and fear-potentiated startle in panic disorder patients. *Biol Psychiatry* 1994; **35**: 431–439.

25 Grillon C, Lissek S, Rabin S, McDowell D, Dvir S, Pine DS. Increased anxiety during anticipation of unpredictable but not predictable aversive stimuli as a psychophysiologic marker of panic disorder. *Am J Psychiatry* 2008; **165**: 898–904.

26 Bailey JE, Dawson GR, Dourish CT, Nutt DJ. Validating the inhalation of 7.5% CO2 in healthy volunteers as a human experimental medicine: a model of generalized anxiety disorder (GAD). *J Psychopharmacol* 2011; **25**: 1192–1198.

27 Golestani AM, Chang C, Kwinta JB, Khatamian YB, Jean Chen J. Mapping the end-tidal CO2 response function in the resting-state BOLD fMRI signal: Spatial specificity, test-retest reliability and effect of fMRI sampling rate. *Neuroimage* 2015; **104**: 266–277.

28 Peng T, Niazy R, Payne SJ, Wise RG. The effects of respiratory CO2 fluctuations in the resting-state BOLD signal differ between eyes open and eyes closed. *Magn Reson Imaging* 2013; **31**: 336–345.

29 Sicard KM, Duong TQ. Effects of hypoxia, hyperoxia, and hypercapnia on baseline and stimulus-evoked BOLD, CBF, and CMRO2 in spontaneously breathing animals. *Neuroimage* 2005; **25**: 850–858.

30 Herrmann MJ, Boehme S, Becker MPI, Tupak S V., Guhn A, Schmidt B *et al.* Phasic and sustained brain responses in the amygdala and the bed nucleus of the stria terminalis during threat anticipation. *Hum Brain Mapp* 2016; **37**: 1091–1102.

31 Alvarez RP, Chen G, Bodurka J, Kaplan R, Grillon C. Phasic and sustained fear in humans elicits distinct patterns of brain activity. *Neuroimage* 2011; **55**: 389–400.

# Supplemental Tables

# Supplemental Figure Captions

**Supplemental Figure 1. Cross-correlation matrices including all variables from the CO2 challenge and NPU sessions. A)** Unthresholded correlation matrix. **B)** Thresholded correlation matrix, corrected for multiple corrections based on permutation tests. Warm colors indicate positive correlations, while cool colors indicate negative correlations. Boxes on the diagonal indicate within-experiment correlations.

**Supplemental Figure 2. Scree plots demonstrating outcome of the principal components analyses for the CO2 challenge and NPU sessions. A)** Scree plot demonstrating outcome of the principal components analyses for the CO2 challenge. **B)** Scree plot demonstrating outcome of the principal components analyses for the NPU laboratory session. **C)** Scree plot demonstrating outcome of the principal components analyses for the NPU fMRI session. Components with an eigenvalue > 1 are considered signal components, while those with an eigenvalue < 1 are considered noise components.

**Supplemental Figure 3. Variability in specific dependent measures accounted for by the signal components in the CO2 and NPU sessions. A)** Variability (r^2^) in dependent measures from the NPU sessions accounted for by the signal components in the CO2 challenge. **B)** Variability (r^2^) in dependent measures from the CO2 challenge and NPU fMRI session accounted for by the signal components in the NPU laboratory session. **C)** Variability (r^2^) in dependent measures from the CO2 challenge and NPU laboratory session accounted for by the signal components in the NPU fMRI session. Filled bars are significant after correcting for multiple comparisons. Hatched bars are trends, but not significant after correcting for multiple comparisons.

**Supplemental Figure 4. Weighted beta coefficients showing contributions of specific items from each experiment to the corresponding PCA/Regression model predicting outcome measures from the remaining experiments. A)** Weighted beta coefficients showing contributions of specific items from the CO2 challenge to the PCA/Regression model predicting anxiety-related dlPFC activity. **B)** Weighted beta coefficients showing contributions of specific items from the CO2 challenge to the PCA/Regression model predicting fear-related dlPFC activity. **C)** Weighted beta coefficients showing contributions of specific items from the NPU laboratory session to the PCA/Regression model predicting subjective responses to the SUDS questionnaire during the CO2 challenge. **D)** Weighted beta coefficients showing contributions of specific items from the NPU laboratory session to the PCA/Regression model predicting respiratory rate (RR) during the CO2 challenge. **E)** Weighted beta coefficients showing contributions of specific items from the NPU fMRI session to the PCA/Regression model predicting fear during the NPU laboratory session. Filled bars represent items from significant PCA/Regression models after correcting for multiple comparisons. Hatched bars represent items from significant PCA/Regression models that are trends, but not significant after correcting for multiple comparisons.

# Supplemental Figure 1

**
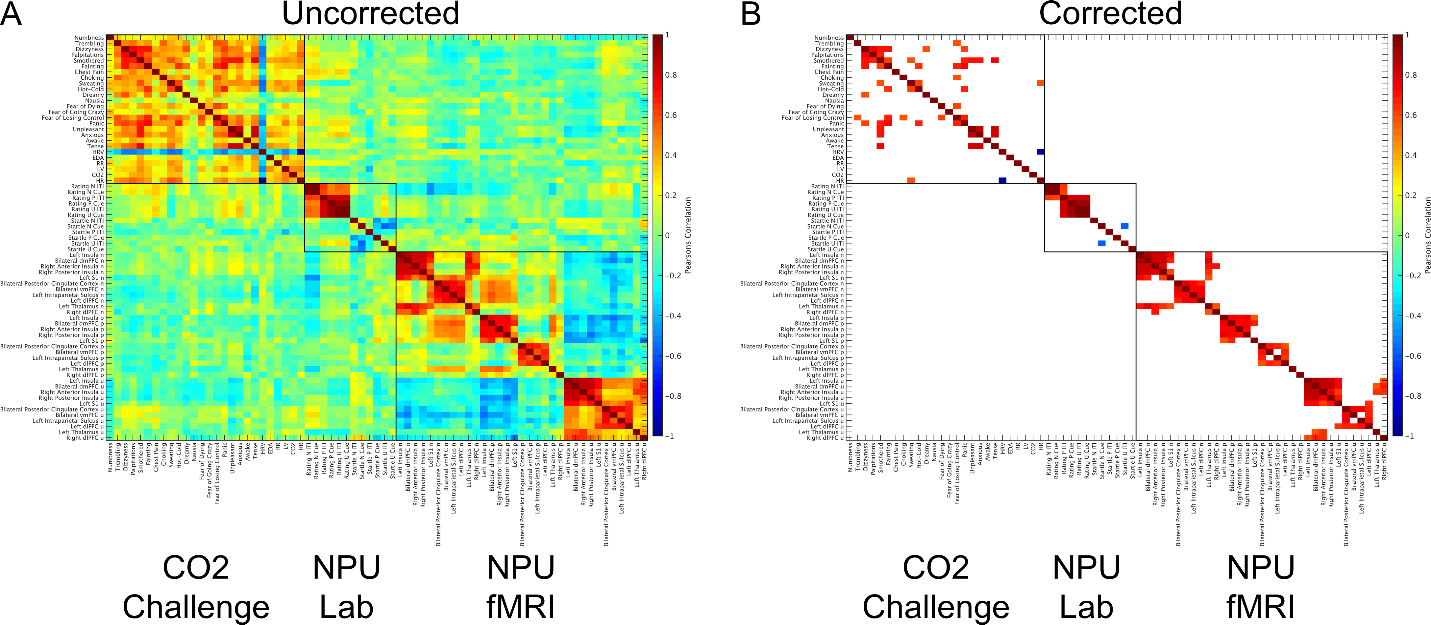
**

# Supplemental Figure 2

**
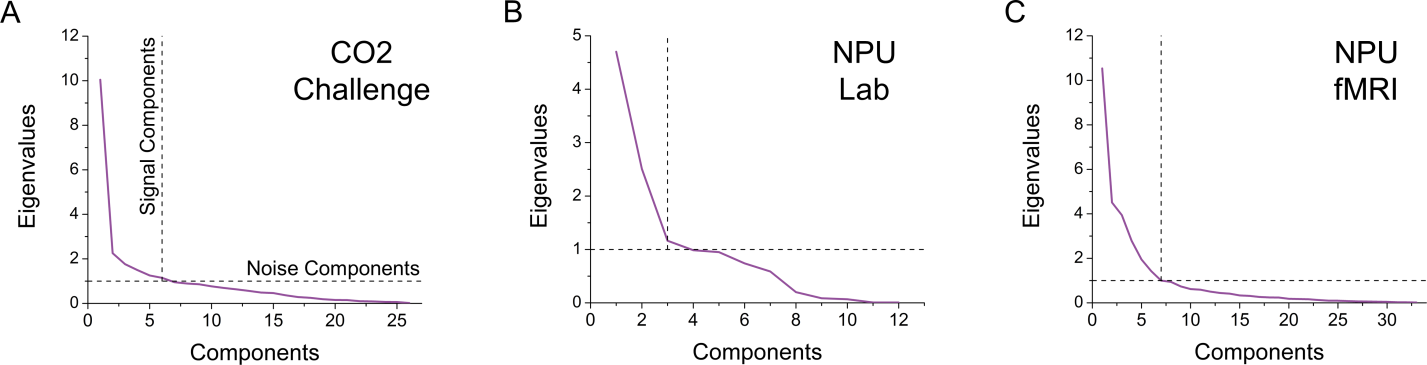
**

# Supplemental Figure 3

**
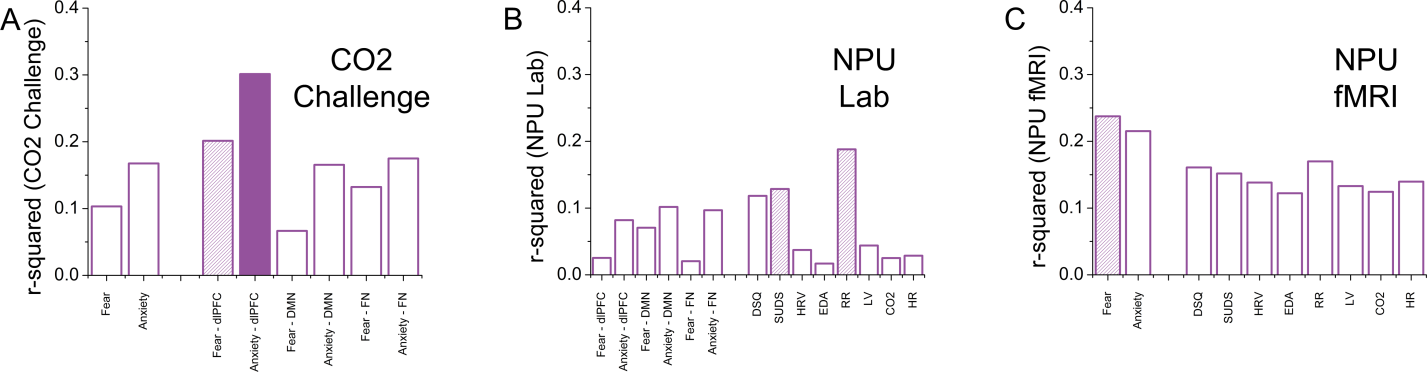
**

# Supplemental Figure 4

**
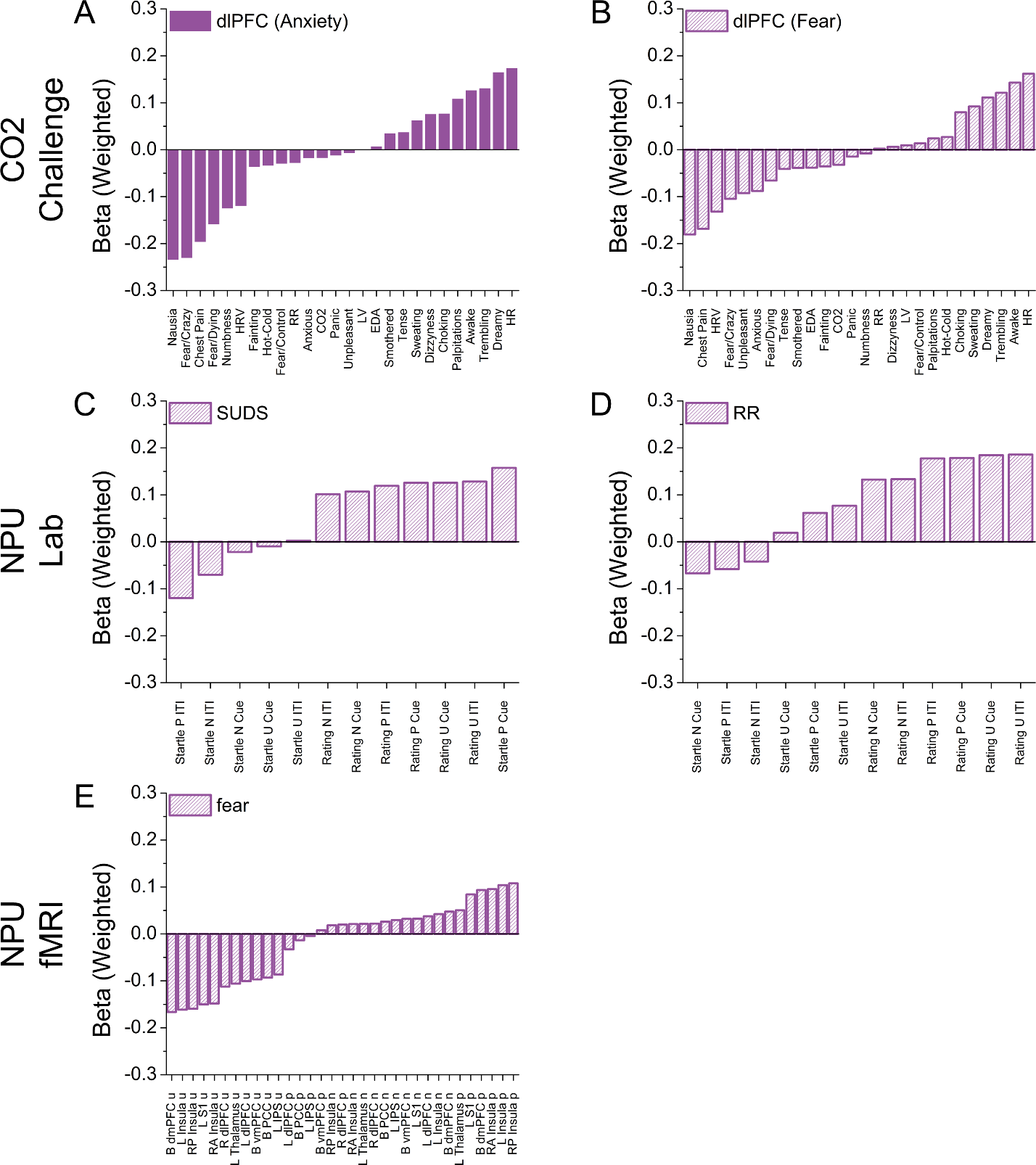
**
